# Glucose Uptake in *Prochlorococcus:* Diversity of Kinetics and Effects on the Metabolism

**DOI:** 10.3389/fmicb.2017.00327

**Published:** 2017-03-08

**Authors:** María del Carmen Muñoz-Marín, Guadalupe Gómez-Baena, Jesús Díez, Robert J. Beynon, David González-Ballester, Mikhail V. Zubkov, José M. García-Fernández

**Affiliations:** ^1^Departamento de Bioquímica y Biología Molecular, Campus de Excelencia Internacional Agroalimentario, Universidad de CórdobaCórdoba, Spain; ^2^Centre for Proteome Research, Institute of Integrative Biology, University of LiverpoolLiverpool, UK; ^3^National Oceanography CentreSouthampton, UK

**Keywords:** marine cyanobacteria, mixotrophy, transporter kinetics, proteomics, gene expression

## Abstract

We have previously shown that *Prochlorococcus* sp. SS120 strain takes up glucose by using a multiphasic transporter encoded by the Pro1404 gene. Here, we studied the glucose uptake kinetics in multiple *Prochlorococcus* strains from different ecotypes, observing diverse values for the K_s_ constants (15–126.60 nM) and the uptake rates (0.48–6.36 pmol min^-1^ mg prot^-1^). Multiphasic kinetics was observed in all studied strains, except for TAK9803-2. Pro1404 gene expression studies during the 21st Atlantic Meridional Transect cruise showed positive correlation with glucose concentrations in the ocean. This suggests that the Pro1404 transporter has been subjected to diversification along the *Prochlorococcus* evolution, in a process probably driven by the glucose availabilities at the different niches it inhabits. The glucose uptake mechanism seems to be a primary transporter. Glucose addition induced detectable transcriptomic and proteomic changes in *Prochlorococcus* SS120, but photosynthetic efficiency was unaffected. Our studies indicate that glucose is actively taken up by *Prochlorococcus*, but its uptake does not significantly alter the trophic ways of this cyanobacterium, which continues performing photosynthesis. Therefore *Prochlorococcus* seems to remain acting as a fundamentally phototrophic organism, capable of using glucose as an extra resource of carbon and energy when available in the environment.

## Introduction

*Prochlorococcus* is one of the main picophytoplankters in the oligotrophic oceans of our planet, and its outstanding ecological importance has been exposed in a wide series of studies published since its discovery (Chisholm et al., 1988; Partensky and Garczarek, 2010; Biller et al., 2015). Given its capability to perform oxygenic photosynthesis, it was considered as a phototrophic organism for a long time (Partensky and Garczarek, 2003) and its ability to utilize organic compounds has often been overlooked. However, different groups have demonstrated that this cyanobacterium can utilize organic compounds, such as amino acids (Zubkov et al., 2003; Michelou et al., 2007; Talarmin et al., 2011; Bjorkman et al., 2015), or dimethylsulfoniopropionate (Vila-Costa et al., 2006). These molecules can be utilized for catabolism and/or anabolism, while providing limiting elements in some cases (as nitrogen or sulfur).

We have previously shown that *Prochlorococcus* can take up glucose at high rates in cultures, inducing specific changes in the expression of genes related to glucose metabolism (Gómez-Baena et al., 2008). More recently, we described that the Pro1404 gene product in *Prochlorococcus* is a glucose transporter with biphasic kinetics and a high affinity constant (K_s_) in the nanomolar range (Muñoz-Marín et al., 2013). Furthermore, we observed that glucose is taken up by natural *Prochlorococcus* populations in the Atlantic Ocean, in environments where glucose is available at nanomolar concentrations (Muñoz-Marín et al., 2013). In good agreement with our findings, some studies have shown that glucose supplementation induces an increase in *Prochlorococcus* populations in the oligotrophic Pacific Ocean (Moisander et al., 2012). However, some essential aspects of this process have not yet been explored. In particular, the mode of glucose transport, how kinetic parameters of the transporter vary across the *Prochlorococcus* radiation, and how glucose uptake affects the physiology of *Prochlorococcus* are not known and could inform the evolutionary history of this process.

In this work, we analyzed the glucose transport kinetics in several strains of *Prochlorococcus* representative of the different ecotypes of this cyanobacterium. We characterized the mode of transport performed by Pro1404, by using several transport and photosynthesis inhibitors. We measured the expression of several genes related to glucose metabolism under low glucose concentrations; in addition, we studied the Pro1404 expression in field samples obtained during the AMT21 cruise. We have also used proteomics to assess how addition of glucose affects *Prochlorococcus* sp. SS120 cultures.

## Materials and Methods

### Seawater Samples Collection

Seawater samples were collected along the Atlantic Meridional Transect 21 cruise (AMT-21), from September 29 to November 14, 2011, at 11 stations (Supplementary Table S1). The seawater was prefiltered through 20 μM mesh and vacuum filtered through 47 mm diameter, 0.2 μm filters (Millipore). Filters were immersed into 4 mL of RNA resuspension buffer (20 mM EDTA; 400 mM NaCl; 0.75 mM sucrose; 50 mM Tris-HCl pH 7) and immediately frozen in liquid nitrogen (Holtzendorff et al., 2002).

### Cyanobacterial Laboratory Strains and Growth Conditions

*Prochlorococcus* spp. strains PCC 9511 [axenic, high-irradiance (HL)-adapted], TAK9803-2 [high-irradiance (HL)-adapted], and SS120, MIT9303, NATL1-A, and NATL2-A [low-irradiance (LL)-adapted] were cultured and collected as previously described (El Alaoui et al., 2001). Cultures were grown in PCR-S11 medium in a culture room set at 24°C under continuous blue irradiances (40 μE/m^2^/s for HL adapted ecotypes and 4 μE/m^2^/s for LL adapted ecotypes) using neon Sylvania F18W/154-ST Daylight, covered with a filter Moonlight blue L183 from Lee Filters.

### Cell Extracts

For proteomic studies, the cells were broken using a French press cell (SLM/Aminco model *FA-079*) at 1,000 psi; the obtained extract was centrifuged for 10 min at 16,000 *g* and 4°C and the supernatant was transferred to a clean tube prior analysis. The cell suspensions stored in Tris buffer were placed in sterile 1.5 mL eppendorf tubes containing glass beads 0.10–0.11 mm of diameter (B. Braun Melsungen AG, Melsungen, Germany). Cell disruption was performed by five cycles of 2 min vortex and 2 min in ice. Tubes were then centrifuged at 16,000 *g* for 5 min at 4°C and the supernant transferred to a new sterile 1.5 mL tube prior analysis.

Protein concentration was determined using the Bio-Rad Protein Assay kit (Bradford, 1976).

### Determination of Glucose Uptake by Cultured Cyanobacterial Strains

[U-^14^C]-glucose (281 mCi/mmol, American Radiolabeled Chemicals, Inc.) was added to 0.7 mL culture samples to reach final concentrations ranging from 0.01 to 20 μM, depending on the experiment. Mixtures of radiolabeled and unlabeled glucose were added to achieve the desired concentration. Glucose uptake, maximum uptake rates and K_s_ constants were determined by the method of Eadie–Hofstee plots (Azam and Hodson, 1981), as previously described (Muñoz-Marín et al., 2013).

### Studies with Inhibitors

[U-^14^C]-glucose was added at a final concentration of 1 μM to a 4.5 mL sample of *Prochlorococcus* SS120 cultures supplemented with different inhibitors (Sigma-Aldrich). Carbonyl cyanide m-chlorophenylhydrazone (CCCP), monensin and valinomicin were disolved in DMSO. *N,N*-dicyclohexylcarbodiimide (DCCD) and (3,4-dichlorophenyl) 1,1 dimethylurea (DCMU) were dissolved in 95% ethanol. 2,5-dibromo-3-methyl-6-isopropyl-*p*-benzoquinone (DBMIB) was prepared in 100% methanol. The final concentrations in the cultures were: 50 μM CCCP, 10 μM monensin, 15 μM valinomycin, 200 μM DCCD, 0.3 μM DCMU, and 0.06 μM DBMIB. All inhibitors were prepared as previously described (El Alaoui et al., 2001; Soontharapirakkul et al., 2011). The effect of ethanol, methanol and DMSO were examined by adding the same concentration to the cultures as above: no substantial alteration in the glucose uptake was noted (not shown).

### Determination of Quantum Yield of Photosystem II

Culture samples (250 mL) were centrifuged at 26,000 *g* for 8 min at 4°C. The pellet was resuspended in 2 mL of PCR-S11 medium and placed in a 24 well culture plate (Biofil). Chlorophyll fluorescence was measured using an IMAGING-PAM chlorophyll fluorometer (M-Series, Maxi version, Heinz Walz GmbH). Cells were dark adapted for 30 min prior to determination of F_o_ and F_m_.

### RNA Isolation from Laboratory *Prochlorococcus* Cultures

RNA was isolated from 500 mL cultures aliquots subjected to either control (standard growth conditions, no glucose added) or glucose conditions (supplemented with 0.1 μM glucose), as described (Domínguez-Martín et al., 2014).

### RNA Isolation from Natural Samples

RNA from natural oceanic samples was isolated with a method modified from that described by Lindell and Post (2001). Filtered seawater samples were mixed with 200 μL of 10% SDS (final concentration 1%) and 18 U of proteinase K (from *Tritirachium album*, Sigma-Aldrich) and incubated at 60°C for 20 min. Then samples were transferred to sterilized *Corex* tubes and 1 mL of phenol was added. Samples were vortexed and centrifuged for 15 min at 15,344 *g*. After two extractions with phenol:chloroform:isoamilic alcohol (25:24:1) and one extraction with chlorophorm:isoamilic alcohol (24:1), in all cases with 15 min centrifugation at 15,344 *g*, 2 mL of 70% isopropanol and 800 μL of ammonium acetate, pH 7.5 were added, and the mixture was kept at -20°C overnight. Samples were centrifuged again for 30 min at 15,344 *g* at 4°C, washed with 70% ethanol, and resuspended in 17 μL of nucleases-free water, 2 μL DNAsa 10x buffer (Ambion) and 2 μL DNAsa (4 U), and incubated for 30–40 min at 37°C. Finally, DNA was removed by addition of 2 μL DNAse Inactivation Reagent. The mixture was incubated for 5 min at room temperature, centrifuged for 5 min at 15,344 *g*, and the supernatant used for further RNA analysis.

### Real-Time Quantitative RT-PCR Analysis of Gene Expression

The synthesis of the cDNA by the reverse transcriptase (RT) reaction was carried out using the iScriptTM cDNA Synthesis kit (Quanta) as recommended by the manufacturers. RNA concentration was quantified using a NanoDrop instrument (Thermo Scientific). For 20 μL of total volume reaction, 0.4 μg of RNA was reverse transcribed for *Prochlorococcus* cultures and 0.1 μg of RNA for field samples. Specific primers to amplify fragments of the the genes of interest from the *Prochlorococcus* strain SS120 were designed using the software Oligo 4.05 (Molecular Biology Insights, Inc.), on the basis of the corresponding *Prochlorococcus* SS120 genome (Dufresne et al., 2003). The primers were tested for *Prochlorococcus* specificity using Primer-BLAST from NCBI. The sequences of the primers used are compiled in **Table 1**.

**Table 1:** List of primers utilized in quantification of gene expression by semi-quantitative RT-PCR.

Gene	Protein	Primer	Sequence
*glcH*	Glucose transporter	Forward	GCTTTTATGGCAGGTTCTTT
		Reverse	CAAATAGCCGCAAGACTCAG
*prk*	Phosphoribulokinase	Forward	ATGGTGGTGTTGTTGGAGAT
		Reverse	CCTTTCAGCA TTGTCACGAT
*fbaA*	Fructose 1-6 biphosphate aldolase	Forward	ATAGGTGAAGAGGTTGGGAG
		Reverse	ATCAGAAGCAGCCAATACAG
*glpA*	Glucose-1P-adenylyltransferase	Forward	TTGCGTTTTAGGCGTTAGAA
		Reverse	TCGTTCCCTGACCTACTCCA
*tal*	Transaldolase	Forward	TATTTGGCTTTTCTCAGGC
		Reverse	GTCCTTCCCTGTTTCTGCT
*gap3*	Glyceraldehyde-3-phosphate dehydrogenase	Forward	GGTAACGCAAGAAGAAGTCA
		Reverse	TGAGTTTTGTTGACCACCAT
*prk*	Pyruvate kinase	Forward	TTCAATTGGTATGGGGTGTA
		Reverse	GGGTTCCTGCTGTTTCTACT
*rbcS*	Ribulose-1,5-bisphosphate carboxylase/oxygenase small subunit	Forward	CCTGGAGACA TTTGGCTT
		Reverse	GTCTGCA TGGAGCCACTT
*rbcL*	Ribulose-1,5-bisphosphate carboxylase/oxygenase large subunit	Forward	TCGCCCTCTTGTTGGTTG
		Reverse	CCTGGCCTGTCTGTTGCT
*rnpB*	RNase subunit B	Forward	TCCCAAAAGACCAGACTTGCT
		Reverse	GTATGTTTCTGTGGCACTCTCCT
*rnpB*	RNase subunit B (used in natural samples; Holtzendorff et al., 2002)	Forward	GGCAAGGGTGCAAAGGTG
		Reverse	GCCGGGGTTTACCTAGCC

As a quality control step, all samples were tested by PCR to check the presence of residual DNA before and after the DNAse treatment. Furthermore, negative control reactions without RT were used to verify the absence of DNA template contamination.

Real time quantitative PCR reactions were performed in triplicate. The reaction mixtures contained 1x of SsoFast^TM^ EvaGreen^®^ Supermix (Bio-Rad), 0.128–0.384 μM forward and reverse primers, depending on the efficiency calculations (not shown) and the corresponding cDNA. The efficiency of the reactions was calculated and optimized following the method described previously (Pfaffl, 2001).

The products were checked for single amplification of DNA fragments of the expected size by agarose gel electrophoresis and by sequencing in the case of seawater samples (not shown). Control reactions were performed in triplicates for each pair of primers in the absence of template.

An iCycler IQ multicolor real time PCR detection system (Bio-Rad) was used for quantitative detection of amplified PCR products using the following thermal cycling conditions: 95°C for 2 min, and 50 cycles of 95°C for 15 s, followed by 58°C for 30 s and 72°C for 30 s. At the end, reactions were checked to discard false amplifications by verifying the melting point of PCR products, determining the fluorescence between 65 and 100°C, with increases of 0.5°C measured each 10 s.

Measurements were carried out in triplicate from at least three independent biological samples subjected to identical culture conditions. The relative change in gene expression was endogenously normalized to that of the gene *rnpB* (**Table 1**), encoding RNase P, calculated using the 2^-ΔΔCt^ method (Pfaffl, 2001). The primers rnpRTfwd/rnpRTrev were used for the endogenous standard (*rnpB*) in the natural samples (Holtzendorff et al., 2002). No change in the expression of *rnpB* was detected under our experimental conditions.

### *Prochlorococcus* Genomic Sequences

Cyanobacterial genomic data were obtained from the Joint Genome Institute^1^^,^^2^ .

### Phylogenetic Analysis

Proteins similar to the glucose transporter Pro1404 were screened within published sequences of *Synechococcus* and *Prochlorococcus* by using the blastp algorithm from the NCBI^3^ imposing a minimum length of 339 amino acids for alignment (>75% of the sequence) and a minimum of 35% identity for *Synechococcus* and 65% identity for *Prochlorococcus* sequences.

GOS sequences were collected by the blastp algorithm against the Pro1404/*glcH* protein from the NCBI in the CAMERA portal^4^ imposing the same restrictions as above. GOS sequences giving a maximum score with *Prochlorococcus* and *Synechococcus* were aligned, using the software SeaView^5^, with the sequences of *Prochlorococcus* and *Synechococcus* detected in the first analysis. Ambiguous areas of alignment were removed using Gblocks (Thermo Scientific), yielding 198 common amino acids. The phylogenetic tree was based on proteins sequences similarity using the maximum likelihood method in the server RaxML BlackBox^6^ under default parameters, using 100 bootstrap replicates and edited by the web-based tool Interactive Tree of Life^7^. The sequences corresponding to *Synechococcus* sp. PCC 7002, *Microcystis aeruginosa, Synechocystis* sp. PCC 6803, and *Thermosynechococcus elongates* BP-1 acted as outgroups in this tree.

### Proteomic Analysis

Cell cultures were grown under standard conditions or in medium supplemented with 0.1 μM glucose for 24 h, before harvesting cells to carry out proteomic analysis. Cell extracts containing 100 μg of protein were incubated with 0.05% (final concentration w/v) of RapiGest (Waters Corporation) for 10 min at 80°C. Samples were then reduced with 3 mM DTT for 10 min at 60°C, followed by carbamidomethylation with 9 mM iodoacetamide for 30 min in the dark at room temperature. Finally, trypsin (ratio 50:1) was added and samples incubated overnight at 37°C. Digestions were stopped by addition of trifluoroacetic acid (TFA) at final concentration of 0.5% (v/v) followed by incubation for 30 min at 37°C. To remove acid inactivated detergent and all insoluble material, sample digests were centrifuged at 13,000 *g* for 15 min. Completeness of digestion was checked by SDS-PAGE (not shown).

Three biological replicates of each condition (control vs. glucose) were used in the analysis, and tryptic digests were analyzed on a QExactive mass spectrometer (Thermo Scientific). Relative and absolute abundances were calculated by a label free approach using Progenesis QI (Waters Corporation). Specifically, the Hi3 method (Silva et al., 2006) was used to gain absolute quantification of the detected proteins. This method is based on the principle that the intensity of the three most intense peptides obtained after trypsin digestion is proportional to the abundance of the protein, and also constant in all proteins, with a 10% error. In our experiments, 50 fmol of rabbit glycogen phosphorylase (Waters Corporation) were added to the sample as internal standard for absolute quantification.

LC–MS/MS analysis was performed using a QExactive instrument (Thermo Scientific) coupled to an Ultimate 3000 chromatography system (Thermo Scientific). Protein digests were trapped on a PepMap 100 C18 precolumn, 300 μm id, 5 mm long, 5 μm particles, 100 Å (Thermo Scientific) for 7 min, at a flow rate of 4 μL/min in 2% (v/v) ACN/0.1% (v/v) TFA, and peptides were separated through a analytical Easy-spray PepMap RSLC C18 column, 75 μm id, 50 cm long, 2 μm particles, 100 Å (Thermo Scientific) at 300 nL/min over a 120 min linear gradient from 3 to 40% (v/v) ACN in 0.1% (v/v) formic acid. The QExactive instrument was operated in data dependent acquisition mode. Full scan MS spectra (m/z 300–2000) and fragment ions were acquired in the orbitrap at 70,000 and 35,000 resolution, respectively. Dynamic exclusion was set to 20 s. The 10 most intense multiply charged ions (*z* ≥ 2) were sequentially isolated and fragmented by high energy collisional dissociation (HCD) at 30% standardized HCD collision energy.

Proteome Discoverer version 1.4 (Thermo Scientific) was used to generate peaklists using default parameters and MASCOT search engine (version 2.4.0, Matrix Science) to identify peptides and proteins, using a database containing all the entries annotated for *Prochlorococcus marinus* sp. SS120 (downloaded on 290415, 1,881 sequences) in Uniprot^8^ and applying a FDR < 1% using the Decoy database tool in MASCOT. Trypsin was selected as specific enzyme allowing one missed cleavage. Carbamidomethylation of cysteines was selected as fixed modification and methionine oxidation as variable, mass tolerance of 10 ppm for precursors and 0.01 Da for fragment ions.

Quantitative data were obtained using Progenesis QI software (Waters Corporation). Features with positive charge states between +2 and +7, and three or more isotopic peaks were taken to further identification. A merged peak list generated by Progenesis QI and searched against the above described database in MASCOT search engine (version 2.4.0, Matrix Science) using the search parameters described above. MASCOT results were imported in Progenesis QI and a peptide cut off score of 20 was applied after manually evaluating the quality of the lowest scored peptides. Hi3 expression data was obtained using the Progenesis QI tool. For this purpose, 50 fmol of glycogen phosphorylase (SwissProt P00489, MassPREP digestion standard, Waters Corporation) were used as internal standard. Similar proteins were grouped and only non-conflictive features (unique peptides) were used for quantification. For a protein to be considered as significantly differentially expressed, it has to be identified and quantified using at least two unique peptides and an ANOVA *p*-value ≤ 0.05.

### Statistical Analysis

Experiments were carried out at least with three independent biological replicates. The results are shown with error bars corresponding to the standard deviation. Significance of data was assessed by using the Student’s *t*-test (^∗^*p* ≤ 0.05; ^∗∗^*p* ≤ 0.01).

## Results and Discussion

### Diversity of Glucose Uptake Kinetics in *Prochlorococcus* Strains

In previous studies, we compared the glucose uptake kinetics of *Prochlorococcus marinus* SS120 with that of *Synechococcus* WH7803, a potential competitor in oligotrophic areas of the oceans (Muñoz-Marín et al., 2013). By analyzing the K_s_ constant and corresponding V_max_, we observed that *Prochlorococcus* was almost seven times more efficient than *Synechococcus* (Muñoz-Marín et al., 2013). In the present work, we assessed possible differences within the *Prochlorococcus* genus, by comparing the glucose uptake kinetics among different strains representative of different evolutive clades: *Prochlorococcus* spp. strains PCC 9511, MIT9303, NATL1A, NATL2A, and TAK9803-2. The strain TAK9803-2 belongs to the recently evolved HL II clade (West et al., 2001). The strains PCC 9511 [genetically identical to MED4 (Rippka et al., 2000)] belongs to the HL I clade (West et al., 2001; Martiny et al., 2009), also late-branching in the *Prochlorococcus* radiation. The strains NATL1A and NATL2A belong to the LL I clade, and SS120 to the LL II/III: both clades occupy an intermediate position in the phylogeny of *Prochlorococus*. Finally, the strain MIT9303 is one of early-branching members of the *Prochlorococcus* radiation and belongs to the LL IV clade (Partensky et al., 1999; West et al., 2001; Martiny et al., 2009). The selected strains cover all *Prochlorococcus* clades with cultured representatives (Biller et al., 2015). We used one axenic strain (PCC 9511, Palinska et al., 2000), while the rest were non-axenic cultures. The fact that the cultures are non-axenic might raise some concern about the results; however, the level of contamination in our cultures is very low (Gómez-Baena et al., 2008); furthermore, we showed that the kinetics of glucose uptake was the same (including both K_s_ value and multiphasic kinetics) when comparing the non-axenic *Prochlorococcus* sp. strain SS120 with an axenic, recombinant strain of *Synechococcus* sp. strain PCC 7942 (naturally incapable of glucose uptake) expressing the *melB* gene from *Prochloroccus* SS120 (Muñoz-Marín et al., 2013). Therefore, the low level of heterotrophic bacteria present in the culture does not interfere in the results and they reflect the actual values of glucose uptake in *Prochlorococcus*.

**FIGURE 1:**
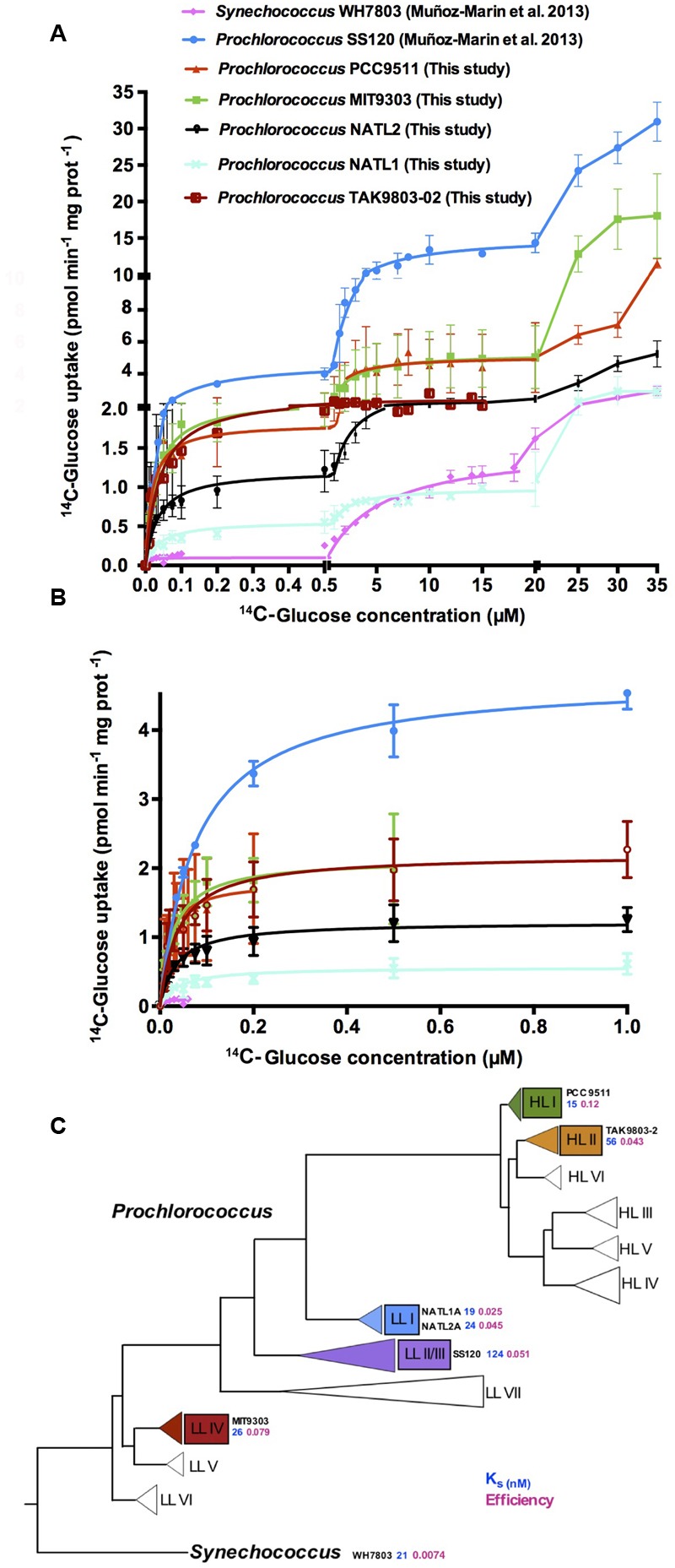
**Kinetics studies of glucose uptake in *Prochlorococcus* sp. strains PCC 9511, MIT9303, NATL1A, NATL2A, and TAK9803-2. (A)** ^14^C-Glucose concentration vs. ^14^C-glucose uptake. **(B)** ^14^C-Glucose concentration vs. its uptake with a different scale in the axis to allow a proper representation of the first phase of the kinetics. Previously described results for SS120 and WH7803 (Muñoz-Marín et al., 2013) are plotted in both graphs as a reference. Data presented are the average of at least three independent biological replicates. Bars show standard deviation for each sample. **(C)** Simplified phylogenetic tree of marine picocyanobacteria based on the rRNA internal transcribed spacer [ITS; adapted from that published by S. W. Chisholm and coworkers (Biller et al., 2015)] showing the evolution of glucose uptake. Numbers next to the name of each strain show the GlcH transporter Ks value (in blue), followed by the efficiency of glucose uptake in pink (as shown in **Table 2**).

Glucose uptake kinetics (maximum uptake rate and K_s_ constant) were determined in the above mentioned strains using laboratory cultures. **Table 2** summarizes the results and **Figures 1A,B** show the kinetics observed for each strain, including in both cases the previously described results for SS120 and WH7803 (Muñoz-Marín et al., 2013) as a reference. **Figure 1C** depicts a simplified phylogenetic tree of the *Prochlorococcus* radiation [adapted from the review by Chisholm and coworkers (Biller et al., 2015)], including also marine *Synechococcus*, showing the K_s_ values and the efficiency for each strain.

**Table 2:** Kinetics parameters in the *Prochlorococcus* sp. strains PCC 9511, MIT9303, NATL1A, NATL2A, and TAK9803-2.

Cyanobacteria		K_s_	V_max_ (pmol min^-1^mg prot^-1^)	Efficiency
*Prochlorococcus* sp.	Phase 1	124.60 ± 7.15 nM	6.36 ± 0.19	0.051 ± 0.0033
SS120 (LL II/III)	Phase 2	2.37 ± 0.87 μM	16.15 ± 1.37	0.007 ± 0.0026
*Prochlorococcus* sp.	Phase 1	15 ± 1.40 nM	1.76 ± 0.51	0.12 ± 0.036
PCC 9511 (HL I)	Phase 2	1.09 ± 0.36 μM	5.23 ± 0.75	0.005 ± 0.0017
*Prochlorococcus* sp.	Phase 1	26 ± 4.22 nM	2.06 ± 0.41	0.079 ± 0.020
MIT9303 (LL IV)	Phase 2	0.92 ± 0.38 μM	5.09 ± 2.77	0.0055 ± 0.0038
*Prochlorococcus* sp.	Phase 1	24 ± 3.45 nM	1.08 ± 0.24	0.045 ± 0.012
NATL2 (LL I)	Phase 2	0.88 ± 0.31 μM	2.40 ± 0.24	0.0027 ± 0.0001
*Prochlorococcus* sp.	Phase 1	19 ± 1.95 nM	0.48 ± 0.09	0.025 ± 0.005
NATL1 (LL I)	Phase 2	1.58 ± 0.17 μM	0.94 ± 0.17	0.0006 ± 0.0001
*Prochlorococcus* sp.	Phase 1	56 ± 5.33 nM	2.41 ± 0.76	0.043 ± 0.014
TAK9803-2 (HL II)				
*Synechococcus* sp.	Phase 1	21.55 ± 1.70 nM	0.16 ± 0.01	0.0074 ± 0.0007
WH 7803	Phase 2	2.86 ± 0.22 μM	1.21 ± 0.18	0.00042 ± 0.00007

*This table includes the previously described results for *Prochlorococcus* sp. SS120 and *Synechococcus* sp. WH7803 as a reference. Efficiency = V_*max*_/K_*s*_.*

We observed some diversity for both the K_s_ (15 nM in PCC 9511 vs. 124.6 nM in SS120) and the V_max_ values (0.16 pmol min^-1^ mg prot^-1^ in WH7803 vs. 6.36 pmol min^-1^ mg prot^-1^ in SS120). When both parameters were taken together in order to calculate the efficiency in the glucose uptake (V_max_ /K_s_), the results ranged from 0.0074 to 0.12 (for WH7803 and PCC 9511, respectively); in other words, *Prochlorococcus* PCC 9511 is 16 times more efficient for glucose uptake than *Synechococcus* WH7803. Interestingly, when we correlated the kinetics parameters of glucose transport to the marine picocyanobacteria phylogeny (**Figure 1C**), we observed an increase in the efficiency of the process, which was highest in late-branching HL I strain PCC 9511, and lowest in *Synechococcus* WH7803. It is worth mentioning that our results only include one *Synechococcus* strain, and that another late-branching strain (TAK9803-2, belonging to the HLII clade) shows a value of efficiency similar to earlier branching groups as LL I and LLII/III (i.e., NATL1A, NATL2A, SS120), and thus this is a proposal which should be considered with caution. Further studies addressing a wider range of strains are required to confirm our hypothesis of the trends toward increased efficiency of glucose uptake in marine picocyanobacteria.

The energetic cost of glucose transport is lower than that of its biosynthesis (Muñoz-Marín et al., 2013). These levels of diversity in the glucose uptake suggest that this process might be important for marine cyanobacterial strains, and therefore an increased competence for the uptake of glucose could be a selective advantage under specific conditions (i.e., extended darkness; Coe et al., 2016), thus in part driving the diversification described above. The trend to efficiency increasing for glucose uptake described above fits nicely with this hypothesis.

The maximum glucose uptake rate was much higher in *Prochlorococcus* sp. SS120 than in other strains. Although SS120 is adapted to live at depth where light availability is low, these environments have higher nutrient concentrations than the surface ocean (Cullen, 2015; Yelton et al., 2016). These results are in good agreement with previous studies, showing that the mixotrophy genes are more abundant in low light-adapted ecotypes of *Prochlorococcus* (Yelton et al., 2016) and with higher amino acid uptake rates than populations close to the surface (Zubkov et al., 2004). This might suggest that glucose uptake at depth might be more advantageous for *Prochlorococcus*, although this requires experimental confirmation in the field.

The results showed multiphasic kinetics in all studied strains except in TAK9803-2 (**Figures 1A,B**). The third phase was not fully characterized due to the high concentrations of glucose required to reach saturation, which make them meaningless from an ecological point of view, since the glucose concentrations observed in the oceans are usually in the nanomolar range (Azam and Hodson, 1981; Rich et al., 1996; Muñoz-Marín et al., 2013). It is not clear yet why TAK9803-2 lacks the biphasic kinetics, despite being quite closely related to PCC 9511 (**Figure 1C**). The loss of multiphasic kinetics might be another step in the evolution of glucose uptake in *Prochlorococcus*, taking into account that TAK9803-2 belongs to one of the late-branching clades (HL II). Future studies will address this possibility.

All strains showed a high affinity K_s_ constant (in the nanomolar range) in the first phase of glucose uptake, suggesting that this is a feature of the Pro1404 transporter from marine cyanobacteria (**Figure 1B** and **Table 2**). Therefore we propose to change the current name of this gene (*melB*, for melibiose transporter) to a more appropriate *glcH*, since we have shown it is a very high affinity (H) glucose transporter (*glc*), with higher affinity for glucose than for other sugars (Muñoz-Marín et al., 2013).

### Expression of *glcH* in Natural Populations of *Prochlorococcus*

We have shown that *Prochlorococcus* takes up glucose in the Atlantic Ocean (Muñoz-Marín et al., 2013). In order to check whether *glcH* expression (previously annotated as *melB*) was also regulated in the natural habitat of *Prochlorococcus*, as it seems to be the case in laboratory cultures (Gómez-Baena et al., 2008), we determined *glcH* expression in samples obtained during the AMT21 cruise.

**FIGURE 2:**
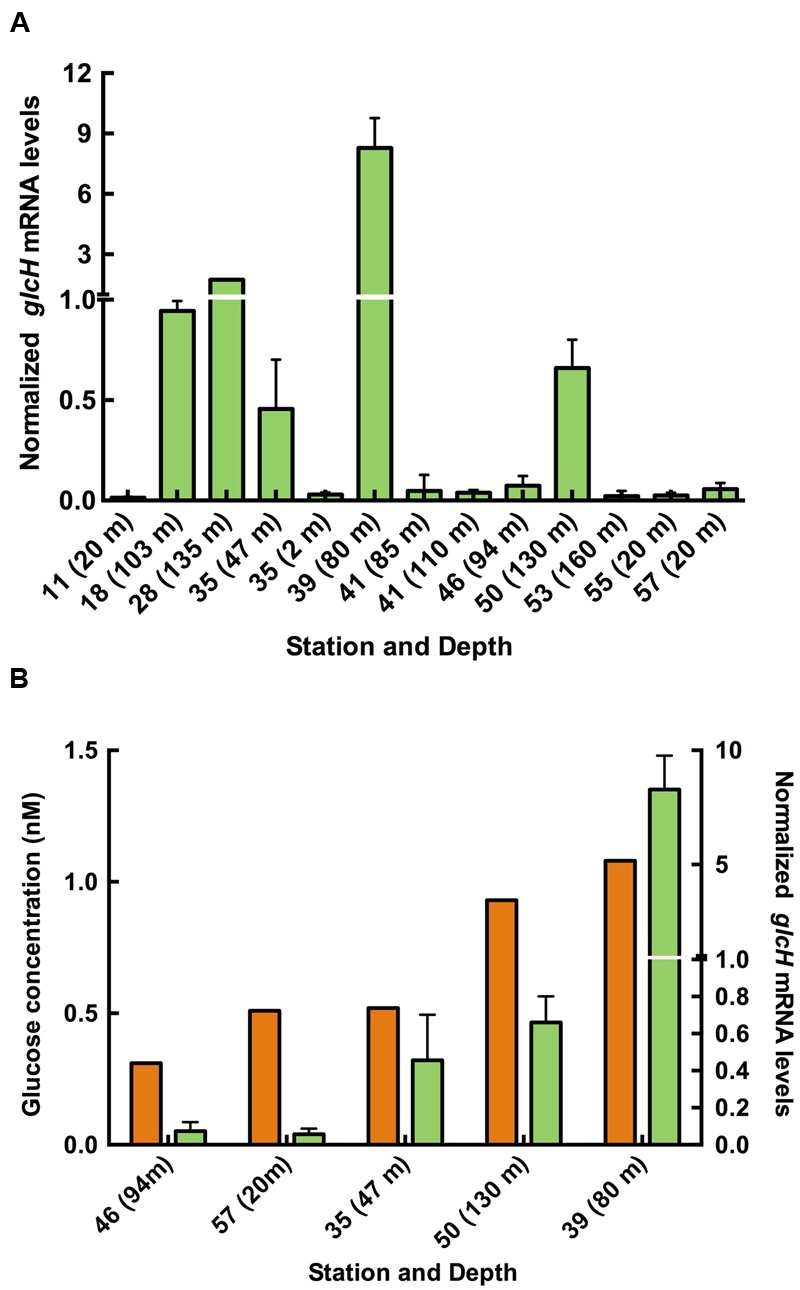
**Expression of *glcH* in natural populations of *Prochlorococcus.* (A)** Relative expression of *glcH* in natural samples of *Prochlorococcus* obtained during the AMT-21 cruise. **(B)** Relationship between the glucose concentration found in the sea (orange) and the *glcH* expression in natural samples of *Prochlorococcus* (green). Information on the specific stations of the AMT21 cruise are available in a previous paper (Muñoz-Marín et al., 2013) and in the AMT21 Cruise Report (http://www.amt-uk.org/pdf/AMT21_Cruise_Report.pdf).

RNA samples were collected at 13 sites^9^ (Supplementary Table S1; Muñoz-Marín et al., 2013), and the *glcH* expression was determined using the same *glcH*/*melB* primers previously utilized for the SS120 strain (**Figure 2A**) (Gómez-Baena et al., 2008). Moreover, we compared the *glcH* expression with the glucose bioavailability and the glucose uptake by *Prochlorococcus* measured during the transect (Muñoz-Marín et al., 2013). Unfortunately, we could only compare the glucose bioavailability to *glcH* expression in five stations, since it was not possible to sample for RNA expression in all stations due to time constraints (**Figure 2B**). We observed that the areas where we found the highest *glcH* expression matched those where *Prochlorococcus* population contributed to maximum glucose transport 2.6–3.7% of the total bacterioplankton glucose uptake (Muñoz-Marín et al., 2013).

The highest *glcH* expression was observed at station 35, 39 and 50 of AMT21, where glucose concentration was higher as well (**Figure 2B**). Our results suggest a positive correlation between glucose concentration and *glcH* expression in *Prochlorococcus*, but this hypothesis requires further studies in the field for confirmation. This is in good agreement with previous results of our team, showing increased *glcH* expression after glucose addition to *Prochlorococcus* sp. SS120 cultures (Gómez-Baena et al., 2008).

### Phylogeny of the Pro1404 Gene

We screened for genes encoding proteins similar to the glucose transporter (*melB*/*glcH*) from *Prochlorococcus* SS120 and used them to build a phylogenetic tree with similar proteins in 74 sequenced strains of *Prochlorococcus* and *Synechococcus* (**Supplementary Figure S1**). To further understand *glcH* transcription in natural samples, we searched in the metagenomic libraries for the glucose transporter gene Pro1404/*glcH* from *Prochlorococcus* to check whether similar genes are often detected in the ocean. The samples used were obtained at the Global Ocean Survey (GOS; covering sites in the Atlantic, Pacific, and Indian Oceans; August 2003–January 2006) (Rusch et al., 2007; Yooseph et al., 2007; Williamson et al., 2008).

The phylogenetic analysis between *Prochlorococcus* and *Synechococcus* sequences revealed three clades: the entire marine *Synechococcus* sequences, which represent clades a and b, and another distinct clade c belonging one sub-clade to the *Synechococcus* genus and the big sub-clade of the *Prochlorococcus* genus. The glucose transporter (*melB*/*glcH*) is highly conserved in the *Prochlorococcus* strains, being present in all of them (Dufresne et al., 2003; Rocap et al., 2003; Biller et al., 2014). This fact, taken together with the variation in the kinetic parameters (**Figure 1A**) and the diversity observed in its phylogeny (**Supplementary Figure S1**), suggests that the *glcH* gene is important for *Prochlorococcus* and is selectively maintained. The large diversity of GOS sequences observed in the HLII clade provide additional support to this hypothesis. Therefore the efficiency in glucose transport, and the mixotrophic capabilities it confers, might be an important factor in the diversification of marine picocyanobacterial ecotypes.

Recent studies addressed the global genetic capacity for mixotrophy in marine cyanobacteria (Yelton et al., 2016), showing a trend to loss genes encoding transporters of organic compounds from the LLIV to the high HLII clades in *Prochlorococcus*. Moreover, the distribution of *glcH* genes in the ocean (**Supplementary Figure S1**, Yelton et al., 2016) is consistent with the presence of the different *Prochlorococcus* and *Synechococcus* ecotypes, in good agreement with our hypothesis. This fact is even more remarkable if we take into account that coastal isolates of the highly abundant SAR11 heterotrophic marine bacteria could utilize glucose as a carbon source, but some open ocean isolates could not (Schwalbach et al., 2010). Furthermore, all *Prochlorococcus* clades have a single glucose transporter gene (*glcH*), except clade LL IV where the presence of *glcP* [a specific glucose permease found in free-living and symbiotic cyanobacteria (Picossi et al., 2013)] has also been reported (Yelton et al., 2016). This reinforces the importance of the high affinity GlcH glucose transporter for *Prochlorococcus*; interestingly, all the strains studied in the sub-cluster 5.1 B of *Synechococcus* possess more than one gene encoding sugar transporter (Yelton et al., 2016).

Another interesting aspect described by Yelton et al. (2016) is that the average number of sugar transporter genes per genome (both in *Prochlorococcus* and *Synechococcus*) is roughly similar in coastal vs. open ocean regions. Therefore, the kinetic diversity described in our work (**Figures 1A,B** and **Table 2**) suggests that selective adaptation might be acting on the efficiency of the sugar uptake (**Figure 1C**), rather than on the number of sugar transporter genes.

### Characterization of the GlcH Transporter with Specific Inhibitors

#### Transport Inhibitors

The energetics of the glucose transport system of *Prochlorococcus* SS120 was also studied. Our initial hypothesis considered that glucose uptake could be most useful for *Prochlorococcus* under energy-limited conditions; however, we have shown that the glucose uptake rate was higher under light, with a circa 40% decrease when cells were subjected to darkness (Gómez-Baena et al., 2008), in accordance with what has been described in previous studies on uptake of organic compounds (Rubin et al., 1977; Church et al., 2004, 2006; Michelou et al., 2007; Mary et al., 2008; Talarmin et al., 2011; Gómez-Pereira et al., 2013; Bjorkman et al., 2015). To evaluate if GlcH is a primary or secondary active transporter, we used different inhibitors of active transport to evaluate if GlcH is a primary or secondary active transporter (i.e., uses energy provided by ATP or from ionic gradients in order to take up glucose, respectively), namely monensin, valinomycin, DCCD, and CCCP.

Monensin is an antibiotic with high selectivity for sodium over other metal cations while valinomycin shows a high selectivity for potassium (Hilgenfeld and Saenger, 1982). The inhibitor DCCD reacts with the carboxyl group of the conserved amino acid residue in the *c*-subunit present in all ATP synthases to inactivate the enzyme (Shoshan and Selman, 1980; Yoshida et al., 1981). CCCP is classified as an uncoupler increasing the proton permeability of the membrane, thereby preventing the formation of the proton gradients needed for ATP synthesis (Mitchell, 1979). Therefore, monensyn and valinomycin are inhibitors of secondary transport, while CCCP and DCCD are inhibitors of primary transport.

**FIGURE 3:**
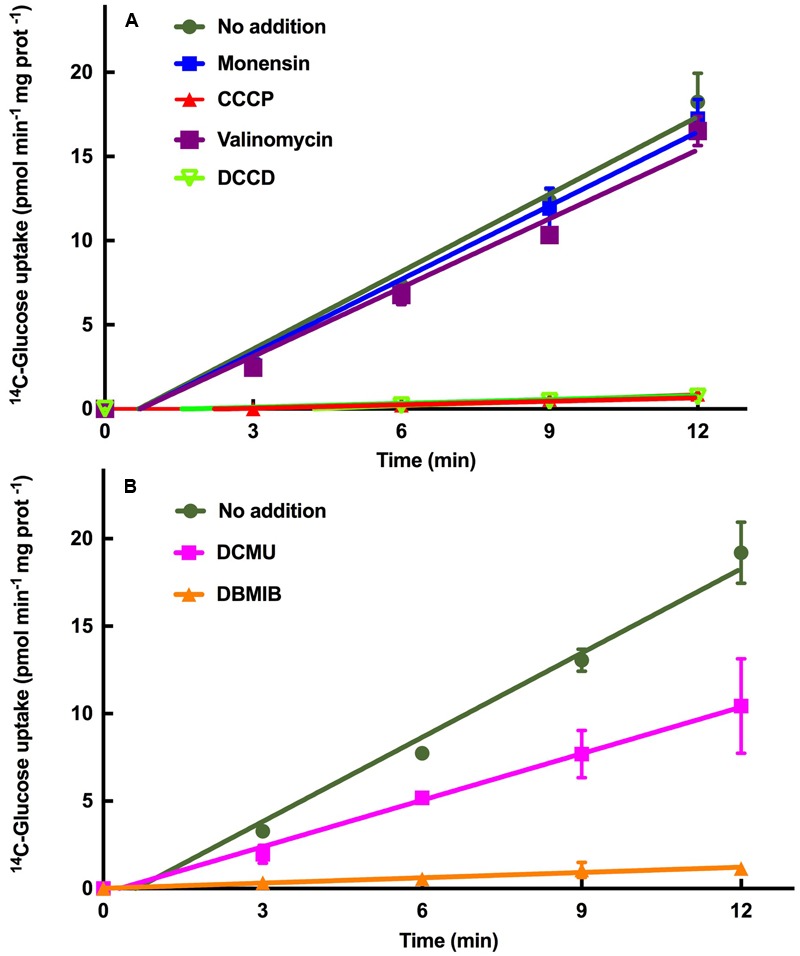
**Characterization of the GlcH transporter of *Prochlorococcus* sp. SS120 with specific inhibitors. (A)** Effect of monensin, CCCP, valinomycin and DCCD on glucose uptake. **(B)** Effect of DCMU and DBMIB on glucose uptake. In both graphs the data are the average of three independent biological replicates. Bars show standard deviation for each sample.

**Figure 3A** shows the results obtained after addition of these inhibitors to *Prochlorococcus* sp. SS120 cultures. Monensin and valinomycin had no effect on the transport, compared to the control values. This suggests that the transport mediated by GlcH is not a symport using either sodium or potassium as ion species. On the other hand, both CCCP and DCCD induced a complete inhibition of glucose uptake, indicating that ATP biosynthesis is essential for this transport, and therefore blocking that process does prevent the uptake of glucose. Therefore, we propose GlcH is an active primary transporter, depending on the generation of ATP to carry out glucose transport. However, the protein encoded by *glcH* has two conserved domains in the sequence (MFS super family and MFS_2 domains), which are typical features of secondary transporters of the Major Facilitator Superfamily (MFS), using Na^+^ as ion (Paulsen et al., 1998; Ethayathulla et al., 2014). The reasons of this discrepancy will be explored in future studies.

The active nature of glucose transport in *Prochlorococcus* (**Figure 3A**) and the fact that glucose uptake is lower in the dark in this organism (Gómez-Baena et al., 2008) fit nicely with the hypothesis proposing that *Prochlorococcus* might invest a significant amount of energy directly from photophosphorylation into organic nutrient acquisition in very oligotrophic environments, probably at the expense of CO_2_ reduction (Zubkov, 2009).

#### Electron Transport Inhibitors

We wanted to explore the participation of photosynthetic electron transport in the glucose uptake regulation. DCMU blocks the transfer of electrons between the PSII and plastoquinone pool (Trebst, 1980) whereas DBMIB prevents electron transfer between the plastoquinone pool and cytochrome b_6_f (Rich et al., 1991). Hence, DCMU allows cyclic electron flow around photosystem I (Bailey et al., 2008; Gao and Wang, 2012).

DBMIB almost completely inhibited the glucose uptake, while DCMU decreased it ca. 50% (**Figure 3B**). The different inhibitory strength of DBMIB and DCMU on the glucose uptake could indicate that the ATP generated by the cyclic electron flow of PS I can maintain up to 50% of the glucose uptake. This is in agreement with previous studies with inhibitors, including marine *Synechococcus* (Bailey et al., 2008), *Prochlorococcus* (Zorz et al., 2015), and other organisms (Gao and Wang, 2012).

### Metabolic Effects of Glucose Uptake in *Prochlorococcus*

To analyze the effects of glucose uptake on *Prochlorococcus* metabolism, we utilized three approaches: first we studied the expression of a number of genes involved in the glucose metabolism in *Prochlorococus* sp. SS120; then we studied the effects on photosynthetic efficiency after glucose addition to cultures of several *Prochlorococcus* strains, and finally we assessed proteomic changes in *Prochlorococcus* sp. SS120. We utilized this strain because it belongs to the low light ecotype, which we expected to be the most benefitted from glucose uptake. For this reason, a significant part of our previous studies on glucose uptake has been carried out in *Prochlorococcus* sp. SS120 (Gómez-Baena et al., 2008; Muñoz-Marín et al., 2013).

#### Effects of Glucose Addition on Gene Expression in *Prochlorococcus* SS120

We have previously described some changes on the gene expression in *Prochlorococcus* sp. SS120 after 1 μM glucose addition (Gómez-Baena et al., 2008). In the present study, we performed 24 h time-course experiments to determine expression of a number of additional genes after addition of 0.1 μM glucose, which is closer to the concentration observed in oligotrophic oceans (Wright and Hobbie, 1966; Azam and Hodson, 1981; Rich et al., 1996; Zubkov et al., 2008; Muñoz-Marín et al., 2013). In order to assess whether glucose availability modifies the main carbon incorporation pathways in *Prochlorococcus*, genes involved in either glucose metabolization or photosynthesis were selected. These included phosphoribulokinase (*frb*), fructose 1,6-biphosphate aldolase (*fbpA*), glucose 1-phosphate-adenylyltransferase (*g1pA*), transaldolase (*tal*), the small and large rubisco (ribulose-1,5-bisphosphate carboxylase/oxygenase) subunits (*rbsSL*), pyruvate kinase (*prk*) and glyceraldehide 3-phosphate dehydrogenase (*g3pA)*.

**FIGURE 4:**
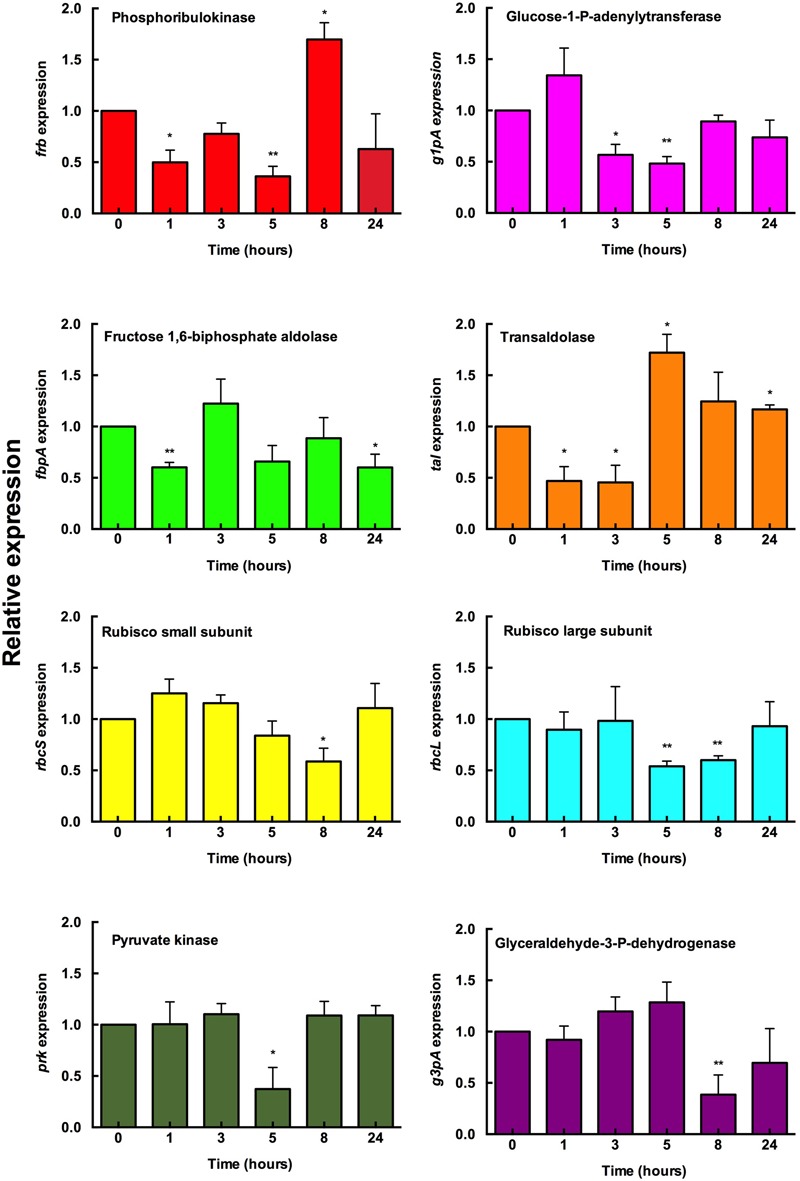
**Effect of glucose addition on the gene expression of *Prochlorococcus* sp. SS120. Glucose (0.1 μM final concentration) was added to *Prochlorococcus* cultures.** Samples were taken at the indicated times and the gene expression measured by real time RT-PCR. Expression level = 1 indicates no changes with respect to the control (no addition). Bars indicate the average of six independent determinations. Error bars show standard deviation for each sample. ^∗^ Means significant with respect to the samples obtained at *T* = 0.

The results were heterogeneous and no major changes in the expression levels were found (**Figure 4**). However, for several genes, an increase in expression was detected after 8 and 24 h (i.e., *frb* and *tal*, respectively); in other cases, a small increase was observed at the beginning of the experiments (i.e., *g1pa, rbcS*).

Interestingly, the Rubisco genes *rbcL* and *rbcS* decreased their expression at 5 and 8 h, while the gene encoding transaldolase (*tal*) increased its expression at those times. This is consistent with a redirection of the flux from the Calvin cycle to the pentoses phosphate pathway; similar changes have been reported in phage-infected *Prochlorococcus* strains (Thompson et al., 2011). Furthermore, this is in agreement with several studies in cyanobacteria and plants which showed that the transcript level of *rbcLS* and other photosynthetic genes decreases under mixotrophic conditions (Quick et al., 1991; Nieva and Fernández Valiente, 1996; Smeekens and Rook, 1997; Yang et al., 2002).

Overall, our data on gene expression suggest that *Prochlorococcus* cells were reacting to the availability of glucose, and probably redirecting to some extent the metabolic fluxes; but the transcriptional response was not being profoundly affected, thus indicating that glucose does not significantly change the trophic strategy of *Prochlorococcus*. This is coherent with the fact that glucose is a central metabolite, and therefore a higher availability of this sugar would not require deep metabolic changes for its utilization.

#### Effects of Glucose Addition on the Photosynthetic Efficiency in Different *Prochlorococcus* Strains

Although expression studies suggested that the transcription of some photosynthetic genes was not affected by glucose addition (**Figure 4**), we addressed whether it might affect the photosynthetic efficiency in different *Prochlorococcus* strains. To this goal, we measured the effective photochemical quantum yield of PSII (Y_(II)_) of *Prochlorococcus* sp. strains SS120, PCC 9511, GP2, NATL1A, NATL2A, MIT9313, and TAK9803-2 cultures 24 h after the supplementation with glucose, and compared to the respective control samples. Y_(II)_ was measured in a range of irradiancies (from 0 to 400 μmol quanta m^-2^ s^-1^) (not shown). These studies did not show any significant change in Y_(II)_ when the cultures were grown in the presence of glucose, suggesting that *Prochlorococcus* retains the functional integrity of its photosynthetic apparatus in the presence of glucose. Nevertheless, in several cyanobacterial species, the heterotrophic growth is known to elicit a number of significant changes in both the structural and functional organization of the photosynthetic apparatus (Evans and Carr, 1975; Smart and Mcintosh, 1991; Vernotte et al., 1992; Mannan and Pakrasi, 1993; Kurian et al., 2006). However, the present study is not the first showing that a cyanobacterium maintains its photosynthetic efficiency during the heterotrophic growth (Raboy et al., 1976).

The presence of glucose might be helpful for *Prochlorococcus* under specific conditions in natural environments. For instance, a recent report has shown that addition of both glucose and pyruvate to *Prochlorococcus* cultures extends their ability to survive prolonged darkness (Coe et al., 2016), although glucose uptake is lower in darkness than in the light (Gómez-Baena et al., 2008). This suggests that other metabolites, such as amino acids (Zubkov et al., 2004), should be considered in addition to glucose to fully understand its physiological effects in *Prochlorococcus*.

#### Effects of Glucose on the Proteome of *Prochlorococcus* sp. Strain SS120

The effect in the proteome profile of 0.1 μM glucose addition to *Prochlorococcus* cultures was measured after 24 h. This time was selected in order to allow the proteome to adapt to the new conditions of glucose availability.

**FIGURE 5:**
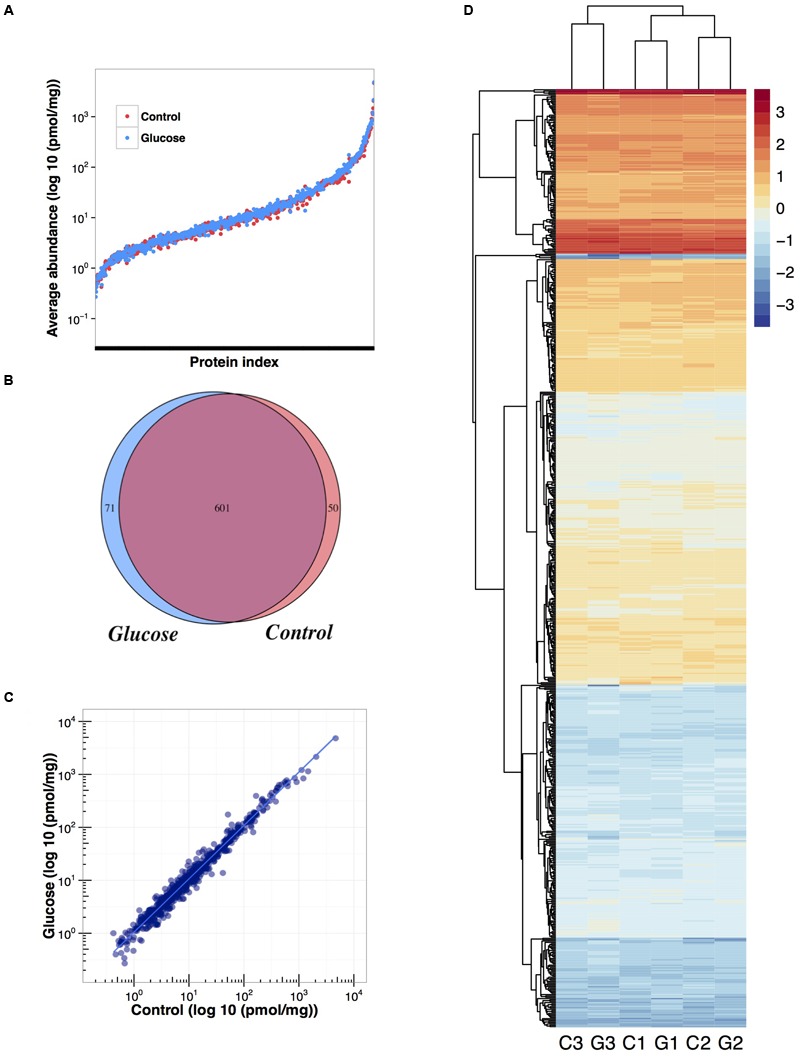
**Effect of glucose addition on the proteome of *Prochlorococcus* sp. SS120: summary of the proteomic analysis. (A)** Overlapping average quantification data obtained for each protein in control and glucose treated cultures. **(B)** Venn diagram showing the number of proteins identified in control and glucose treated cultures. **(C)** Correlation between the average quantification obtained per protein for control and glucose treated cultures. **(D)** Heat map of protein expression (log 10) and hierarchical clustering analysis: C1, control condition from biological replicate 1; G1, glucose condition from biological replicate 1; C2, control condition from biological replicate 2; G2, glucose condition from biological replicate 2; C3, control condition from biological replicate 3; G3, glucose condition from biological replicate 3.

A total of 722 proteins were identified: 601 common to glucose and control conditions, 50 proteins were identified uniquely in the control, and a total of 71 proteins uniquely in the presence of glucose [applying false discovery rate (FDR) < 1% and a least two peptides per identification; **Figure 5** and Supplementary Table S2]. Quantification data was confidently obtained for 702 proteins (Supplementary Table S3).

**FIGURE 6:**
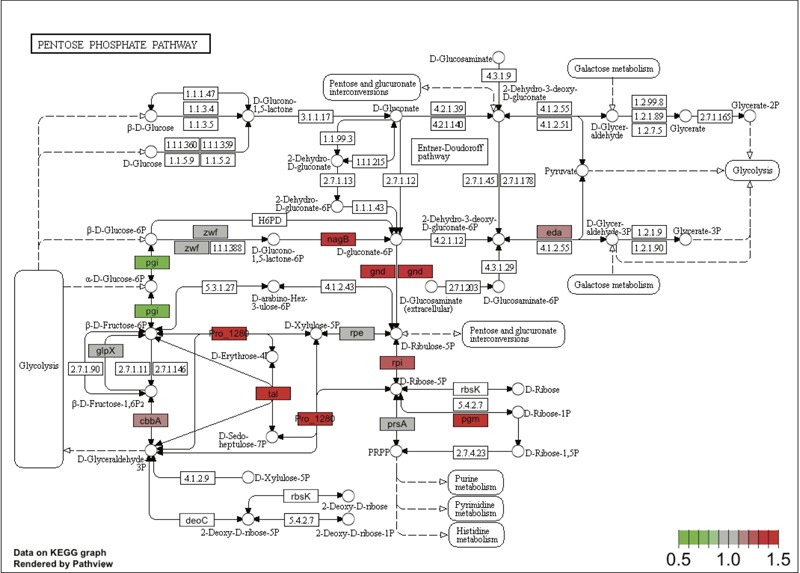
**Changes in the concentration of enzymes involved in glucose utilization in *Prochlorococcus* SS120 upon addition of 0.1 μM glucose.** The scheme depicts the main pathways of glucose utilization in *Prochlorococcus* SS120 using the KEGG database, showing the detected changes in concentration for the different enzymes. Colors show the fold change (glucose/control) obtained from relative quantitation. Red corresponds to upregulation, green to downregulation.

The addition of 0.1 μM glucose did not promote big changes in the proteome of *Prochlorococcus* (**Figure 6** and **Supplementary Figure S2**), in good agreement with our gene expression results. Most of the proteins remained unchanged under glucose addition. When clustering analysis was performed it was evident that samples from the same biological replicate are grouped together, supporting the fact that no major changes are promoted after glucose addition (**Figure 5D**).

**Figure 6** outlines the pathways of glucose metabolization in *Prochlorococcus* SS120 with the main observed changes in enzyme concentrations. Glycolysis is not active due to the absence of phosphofructokinase. Therefore, glucose can be metabolized either by the Entner-Doudoroff or by the pentoses phosphate pathways. We observed an increase in a number of enzymes belonging to both pathways: in the Entner-Doudoroff pathway, several enzymes showed increases of around 20% while in the pentoses phosphate pathway, three of them showed increases of 30–40%. Namely, 2-keto-3-deoxy-6-phosphogluconate aldolase (which is the key enzyme of the Entner-Doudoroff pathway) increased 20%; on the other hand 6-phosphogluconate dehydrogenase (catalyzing the first specific step in the pentose phosphate pathway) increased 32%. Although these changes might not seem dramatic, it has been described in *Prochlorococcus* that small changes in protein level can drive significant redirection in metabolic fluxes (Waldbauer et al., 2012).

A recent study proposed that *Prochlorococcus* might use the Entner-Doudoroff pathway to metabolize glucose under mixotrophic conditions (Chen et al., 2016). Our results provide support for this hypothesis (**Figure 6**), but also indicate an increase in some enzymes related to the pentose phosphate pathway, in good agreement with our results on gene expression (**Figure 4**). Our results suggest that availability of glucose induces a metabolic redirection of fluxes in *Prochlorococcus*, which would involve obtaining reducing power via the pentoses phosphate pathway, and the synthesis of ATP via the Entner-Doudoroff pathway.

Network analysis was performed using STRING^10^ on other proteins significantly changed after glucose addition (**Supplementary Figure S3**). The results emphasized the connection between three of them related to ribosomal machinery. There was no evidence for a decrease in the concentration of proteins related to CO_2_ fixation (Supplementary Table S3).

These results are consistent with the view that *Prochlorococcus* uses glucose as an extra source of energy and carbon but does not repress photosynthetic CO_2_ fixation. This is in contrast with the results shown in *Synechocystis* PCC 6803 (Kurian et al., 2006) in which the photosynthetic capacity in the presence of glucose was markedly reduced, suggesting a significant down-regulation of CO_2_-fixation during heterotrophic growth. However, it should be noted that the glucose concentration used in the latter study (5 mM) was much higher than that used in our experiments, and cells were kept in darkness.

Several photosynthetic proteins (as Psb27) showed an increase in concentration after 24 h of glucose addition (**Supplementary Figure S4** and Table S3). The increase in proteins involved in photosynthesis might be related to the energy demand for the functioning of the transporter GlcH, which is ultimately provided by photosynthesis. This supports the hypothesis that *Prochlorococcus* is essentially a phototroph organism that uses glucose as an additional source of energy and carbon. This is consistent with the the normal functioning of the photosynthetic apparatus. The regulatory proteins NtcA and P_II_ did not show a significant increase after 24 h in the presence of glucose, in agreement with the expression results previously described (Gómez-Baena et al., 2008). This might be due to a rapid metabolization of glucose taken up in the cell, preventing internal shifts in C/N ratios.

**FIGURE 7:**
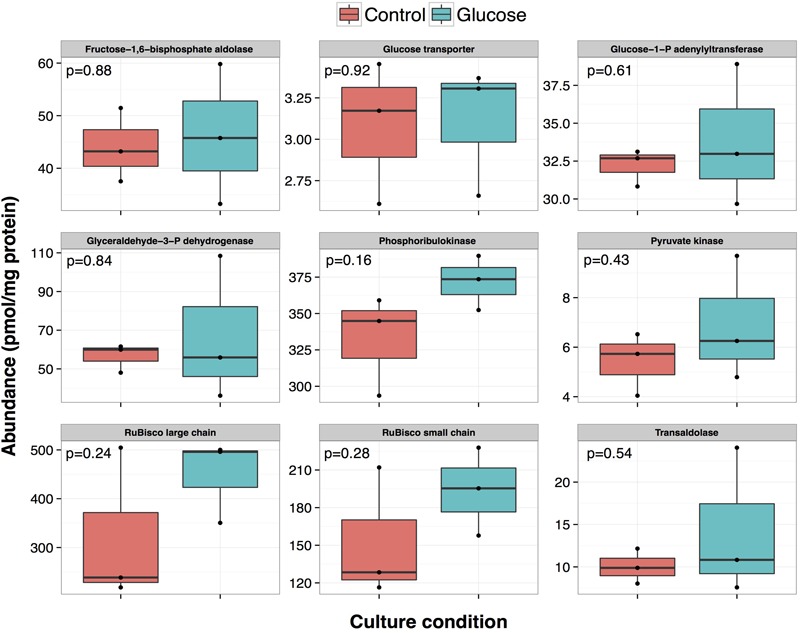
**Effect of glucose addition on the proteome of *Prochlorococcus* sp. SS120: effect on the abundance of proteins related to glucose metabolism.** Absolute quantification data obtained for proteins related to glucose metabolism: orange, control condition, green, glucose condition. Corresponding *p*-value is shown in each panel.

There is in general a good correlation if the data derived from proteomics and mRNA quantification are compared (**Figures 4, 7**). For example, in the case of the enzymes phosphoribulokinase and transaldolase, the corresponding gene expressions increased after 5 and 8 h after glucose addition, going back to basal levels after 24 h. Proteomics results are consistent with these data: higher average concentration in glucose treated cultures at 24 h, although this difference was not significant (*p*-value > 0.05).

## Conclusion

In this work we have explored different aspects of glucose uptake in *Prochlorococccus*, such as its kinetic and phylogenetic diversity, the transport mechanism and the effects on the cell metabolism. Our results showed that a significant level of variation exists in the kinetics of glucose uptake in marine cyanobacteria, therefore suggesting that the capability for efficient glucose uptake might be an important feature conferring selective advantages in the oligotrophic oceans. This advantage might be possessed by select members of broadly defined ecotypic groups. The features of the transport mechanism indicate a primary active transport, thus involving the consumption of metabolic energy in order to incorporate glucose. Finally, gene expression and proteomic studies point out to a redirection in some metabolic fluxes, while photosynthetic efficiency remained mostly unchanged. This suggests that *Prochlorococcus* is actively taking up glucose, when available in the environment, as a way of saving metabolic resources to synthesize sugars; but it does not alter its main role as an organism fundamentally autotrophic.

## Author Contributions

MM-M and GG-B performed research; MM-M, GG-B, JD, RB, DG-B, MZ, and JG-F designed research, analyzed data, wrote and approved the final version of the manuscript.

## Conflict of Interest Statement

The authors declare that the research was conducted in the absence of any commercial or financial relationships that could be construed as a potential conflict of interest.

## Footnotes

1 http://genome.jgi-psf.org

2 http://cyano.genome.ad.jp/

3 http://ncbi.nlm.nih.gov

4 http://camera.calit2.net/index.shtm

5 http://pbil.univ-lyon1.fr/software/seaview.html

6 http://embnet.vital-it.ch/raxml-bb/

7 http://itol.embl.de

8 http://www.uniprot.org

9 http://www.amt-uk.org/pdf/AMT21_Cruise_Report.pdf

10 http://string-db.org

## Supplementary Material

FIGURE S1 **Maximum likelihood phylogenetic tree of *glcH* based on protein sequence similarity with the *Prochlorococcus, Synechococcus* and GOS sequences.** The tree includes 55 sequences (shown by their NCBI accesion numbers in the Supplementary Table S1) of *Prochlorococcus* and *Synechococcus* strains and 30 GOS sequences. Bootstrap values (100 replicates) above 75% are indicated on the branches. The sequences corresponding to *Synechococcus* sp. PCC 7002, *Microcystis aeruginosa, Synechocistis* sp. PCC 6803 and *Thermosynechococcus elongates* BP-1 acted as outgroups in this tree.

FIGURE S2 **Correlation plot between the relative protein abundance (log 10) obtained from individual samples.** Bottom panels show the correlation scatter plot and top panel show the correlation coefficient.

FIGURE S3 **Network illustrating the relationship between proteins significantly changed after glucose addition.** Proteomics was used to evaluate the effect of glucose addition on the proteome of *Prochlorococcus* SS120. Analysis performed using String (http://string-db.org) requiring low confidence (0.150) and adding two (white) nodes. Different colors represent different types of evidence for association: (green) Neighborhood, (red) Gene fusion, (navy blue) Coocurrence, (black) Coexpression, (pink) Experiments, (light blue) Database.

FIGURE S4 **Effect of glucose addition on the proteome of *Prochlorococcus* sp. SS120: proteins significantly changed after glucose addition.** Each panel shows the absolute quantification data obtained for proteins significantly changed (*p*-value < 0.05): orange, control condition, green, glucose condition. Corresponding *p*-value is shown in each panel.
