# Supplementary figures and images for: Glucose Uptake in Prochlorococcus: Diversity of Kinetics and Effects on the Metabolism
Source: Front Microbiol. 2017 Mar 8;8:327. doi: 10.3389/fmicb.2017.00327 (PMC5340979; doi:10.3389/fmicb.2017.00327)

Tree scale: 1

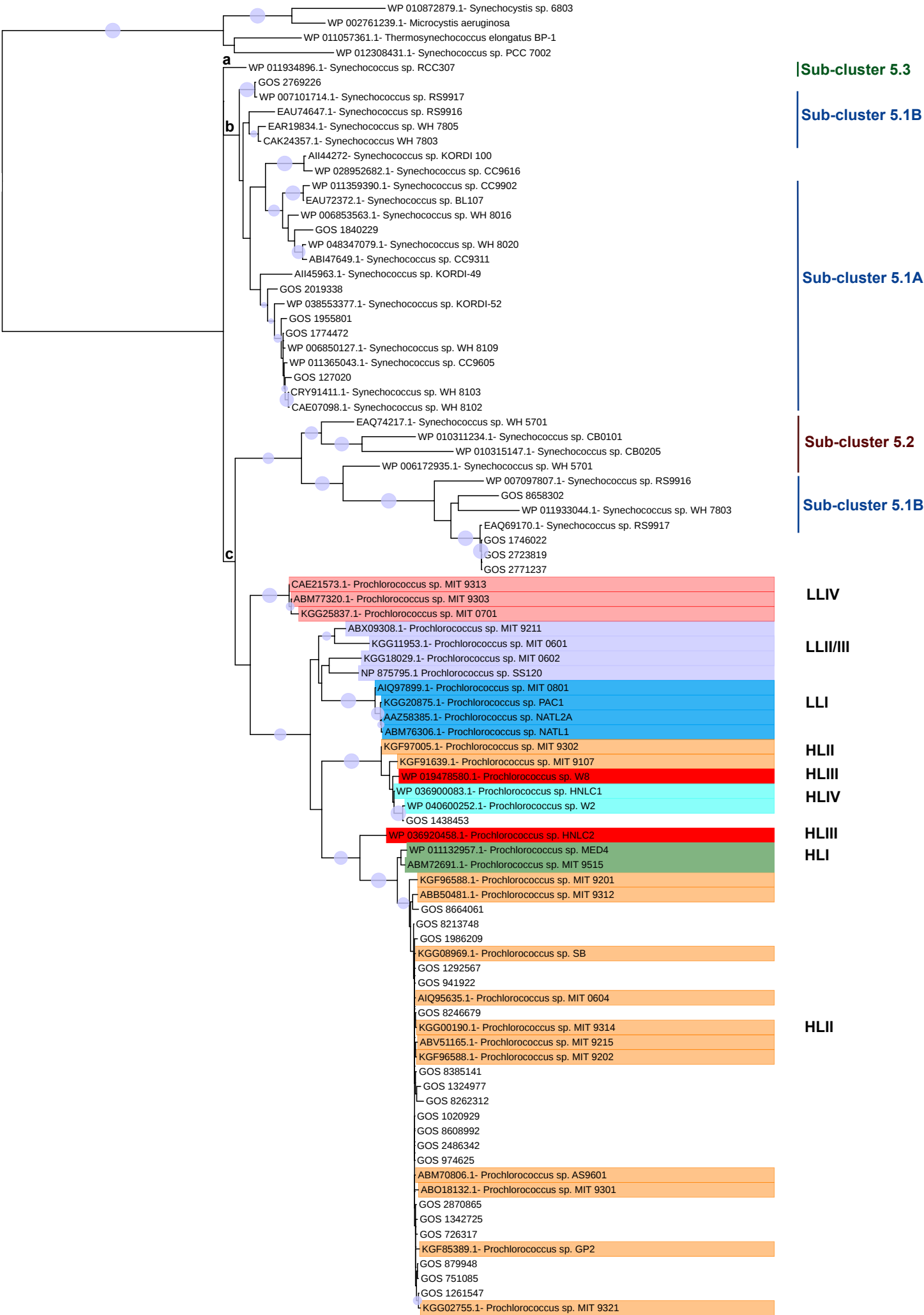

Supplement: FIGURE S1 — Maximum likelihood phylogenetic tree of glcH based on protein sequence similarity with the Prochlorococcus, Synechococcus and GOS sequences. The tree includes 55 sequences (shown by their NCBI accesion numbers in the Supplementary Table S1) of Prochlorococcus and Synechococcus strains and 30 GOS sequences. Bootstrap values (100 replicates) above 75% are indicated on the branches. The sequences corresponding to Synechococcus sp. PCC 7002, Microcystis aeruginosa, Synechocistis sp. PCC 6803 and Thermosynechococcus elongates BP-1 acted as outgroups in this tree. [file Image_1.PDF]

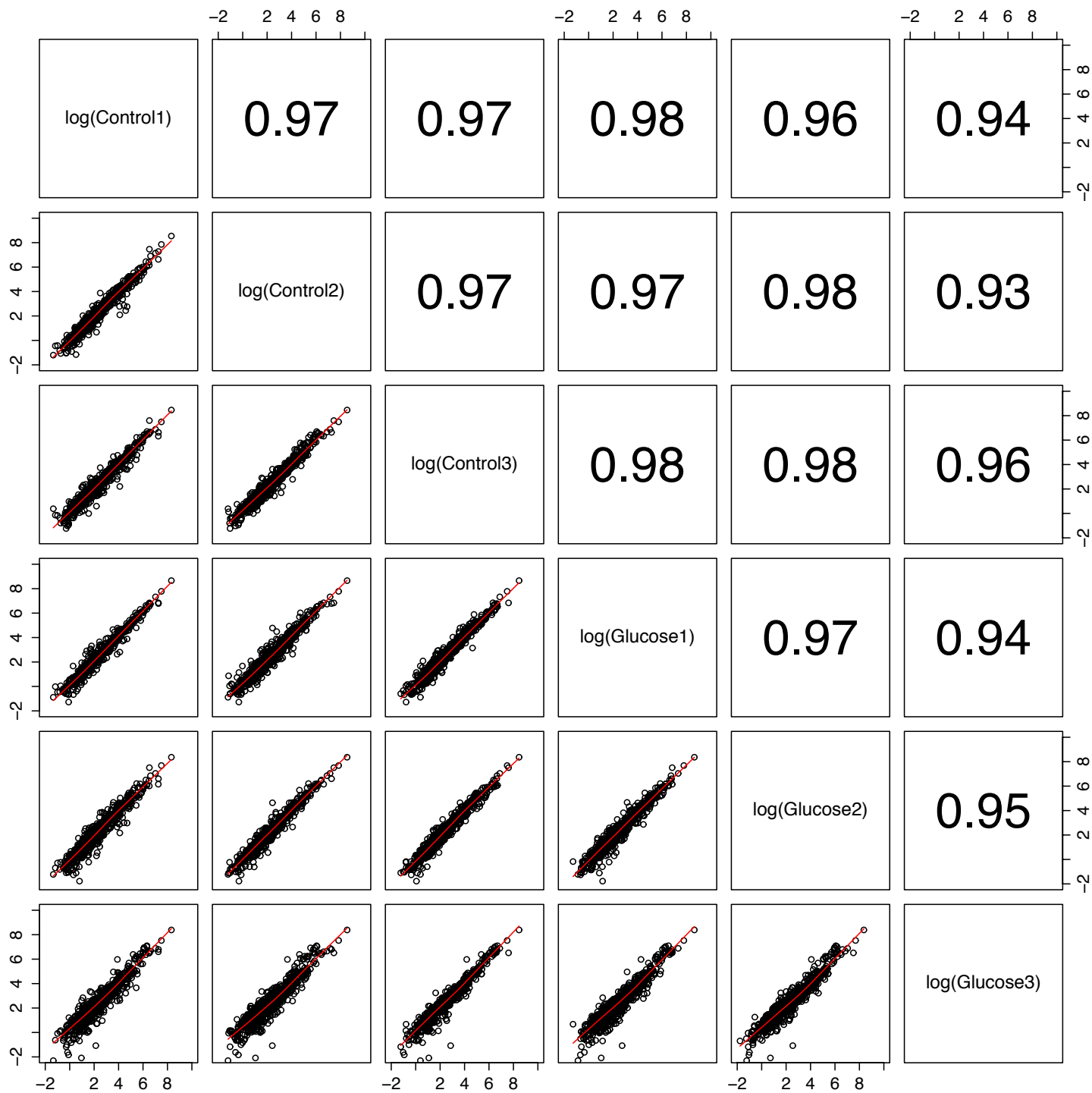

Supplement: FIGURE S2 — Correlation plot between the relative protein abundance (log 10) obtained from individual samples. Bottom panels show the correlation scatter plot and top panel show the correlation coefficient. [file Image_2.PDF]

Abundance (pmol/mg protein)

Control Glucose

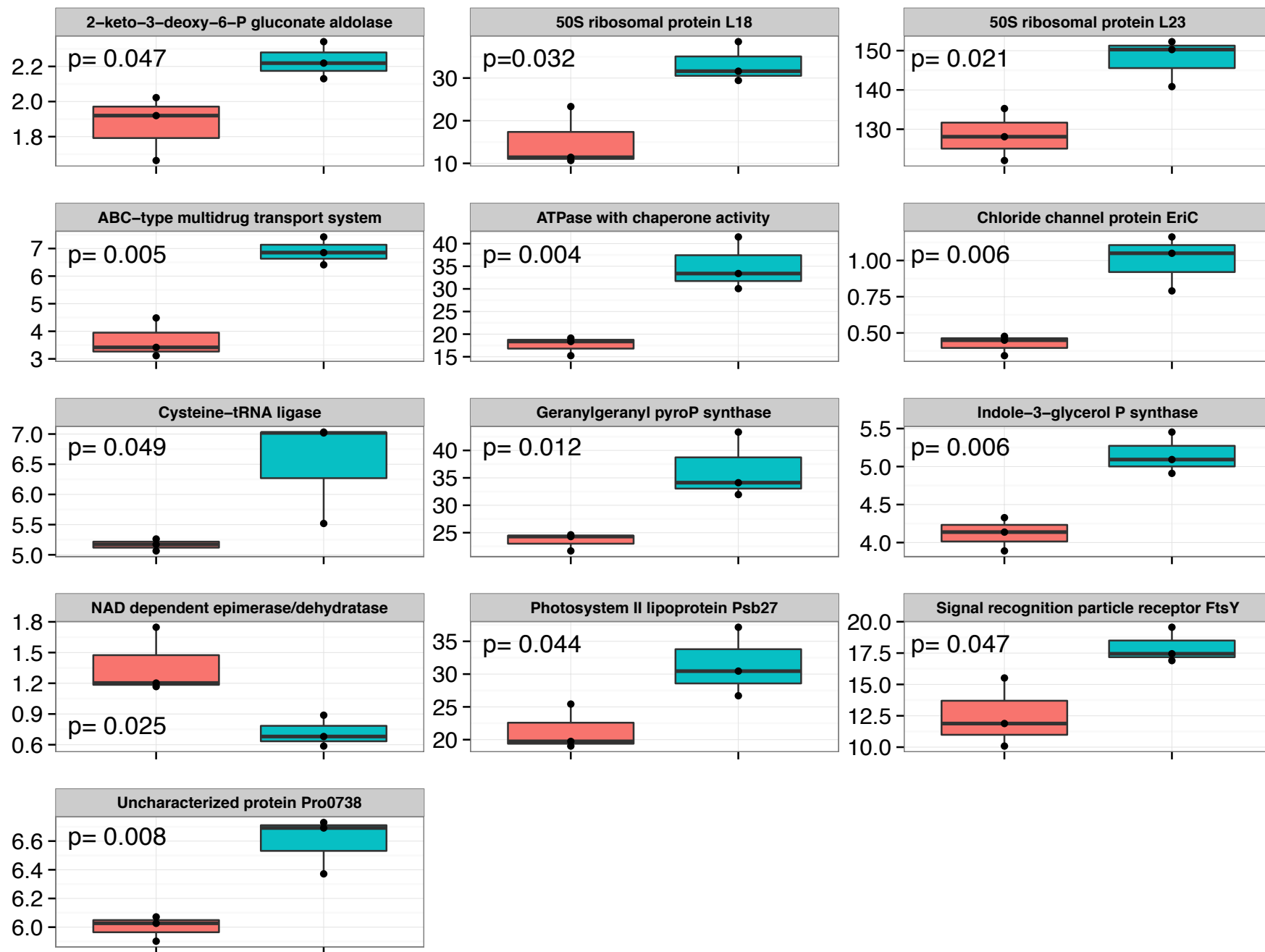

Culture condition

Supplement: FIGURE S4 — Effect of glucose addition on the proteome of Prochlorococcus sp. SS120: proteins significantly changed after glucose addition. Each panel shows the absolute quantification data obtained for proteins significantly changed (p-value < 0.05): orange, control condition, green, glucose condition. Corresponding p-value is shown in each panel. [file Image_4.PDF]
