# Supplementary material for: Glucose Uptake in Prochlorococcus: Diversity of Kinetics and Effects on the Metabolism
Source: Front Microbiol. 2017 Mar 8;8:327. doi: 10.3389/fmicb.2017.00327 (PMC5340979; doi:10.3389/fmicb.2017.00327)
Supplement: FIGURE S3 — Network illustrating the relationship between proteins significantly changed after glucose addition. Proteomics was used to evaluate the effect of glucose addition on the proteome of Prochlorococcus SS120. Analysis performed using String (http://string-db.org) requiring low confidence (0.150) and adding two (white) nodes. Different colors represent different types of evidence for association: (green) Neighborhood, (red) Gene fusion, (navy blue) Coocurrence, (black) Coexpression, (pink) Experiments, (light blue) Database. [file Image_3.PDF]

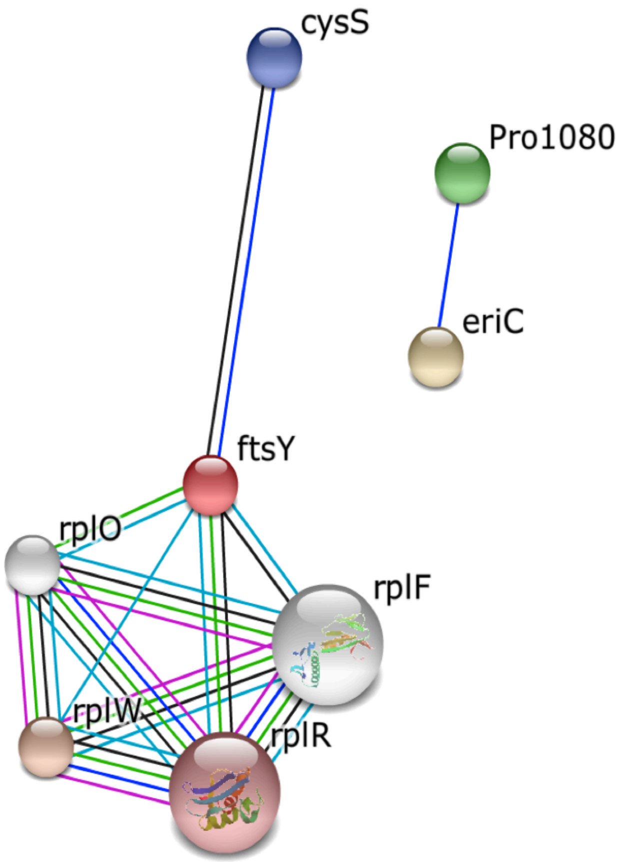

cysS (Cysteine-tRNA ligase)

Pro1080 (NAD dependent epimerase/dehydatase)

eriC (chloride channel protein EriC)

ftsY (Signal recognition particle receptor FtsY)

rplW (50S ribosomal protein L18)

rplR (50S ribosomal protein L23)

Supplementary figure 3: Network illustrating relatioship between proteins significantly changed after glucose addition. Proteomics was used to evaluate the effect of glucose addition on the proteome of *Prochlorococcus* SS120. Analysis performed using String (http://string-db.org) requiring low confidence (0.150) and adding two (white) nodes. Different colors represent different types of evidence for association: (green) Neighborhood, (red) Gene fusion, (navy blue) Coocurrence, (black) Coexpression, (pink) Experiments, (light blue) Database.
