# Supplementary material for: Glucose Uptake in Prochlorococcus: Diversity of Kinetics and Effects on the Metabolism
Source: Front Microbiol. 2017 Mar 8;8:327. doi: 10.3389/fmicb.2017.00327 (PMC5340979; doi:10.3389/fmicb.2017.00327)
Supplement: Supplementary file 5 [file Table_1.DOCX]

**Supplementary Table 1. Station sampled for RNA**

| **Station** | **Geographical coordinates** | **Depths sampled (m)** |
| --- | --- | --- |
| 11 | 38° 30.91' N 23° 07.63' W | 20 |
| 18 | 30°52.871N 32°11.715W | 103 DCM |
| 28 | 20° 23.43' N 38° 33.00' W | 135 DCM |
| 35 | 10° 45.43' N 31° 52.40' W | 2, 47 DCM |
| 39 | 04° 37.59' N 27° 55.16' W | 80 DCM |
| 41 | 01° 55.70' N 26° 13.20' W | 85 DCM, 110 |
| 46 | 07° 42.52' S 25° 02.34' W | 94 DCM |
| 50 | 13° 34.85' S 25° 04.09' W | 130 DCM |
| 53 | 16° 57.62' S 25° 05.65' W | 160 DCM |
| 55 | 20° 00.85' S 25° 05.46' W | 20 |
| 57 | 23° 04.58' S 25° 03.43' W | 20 |
|  |  |  |
